# The Development of Mechanical Allodynia in Diabetic Rats Revealed by Single-Cell RNA-Seq

**DOI:** 10.3389/fnmol.2022.856299

**Published:** 2022-05-20

**Authors:** Han Zhou, Xiaosheng Yang, Chenlong Liao, Hongjin Chen, Yiwei Wu, Binran Xie, Fukai Ma, WenChuan Zhang

**Affiliations:** Department of Neurosurgery, Ninth People Hospital Affiliated to Shanghai Jiao Tong University School of Medicine, Shanghai, China

**Keywords:** mechanical allodynia, neuropathic pain, painful diabetic peripheral neuropathy, somatosensory neurons, single-cell RNA sequencing

## Abstract

Mechanical allodynia (MA) is the main reason that patients with diabetic peripheral neuropathy (DPN) seek medical advice. It severely debilitates the quality of life. Investigating hyperglycemia-induced changes in neural transcription could provide fundamental insights into the complex pathogenesis of painful DPN (PDPN). Gene expression profiles of physiological dorsal root ganglia (DRG) have been studied. However, the transcriptomic changes in DRG neurons in PDPN remain largely unexplored. In this study, by single-cell RNA sequencing on dissociated rat DRG, we identified five physiological neuron types and a novel neuron type MAAC (*Fxyd7*^+^*/Atp1b1*^+^) in PDPN. The novel neuron type originated from peptidergic neuron cluster and was characterized by highly expressing genes related to neurofilament and cytoskeleton. Based on the inferred gene regulatory networks, we found that activated transcription factors *Hobx7* and *Larp1* in MAAC could enhance *Atp1b1* expression. Moreover, we constructed the cellular communication network of MAAC and revealed its receptor-ligand pairs for transmitting signals with other cells. Our molecular investigation at single-cell resolution advances the understanding of the dynamic peripheral neuron changes and underlying molecular mechanisms during the development of PDPN.

## Introduction

Diabetic peripheral neuropathy (DPN) is a common complication of diabetes with variable clinical presentations. Patients can suffer from a painless syndrome with loss of sense of touch and temperature, or neuropathic pain manifested by mechanical allodynia (MA) (Berti-Mattera et al., 2008). The latter is the main reason for patients to seek medical advice as it severely debilitates patients' life quality (Quattrini and Tesfaye, 2003). The pathogenesis of painful DPN (PDPN) is complex. Diabetes can lead to multiple damage to the peripheral nervous system, including abnormal activation of multiple metabolic pathways and imbalance of mitochondrial redox state (Feldman et al., 2017). In addition, nerve swelling caused by metabolic disturbances can result in nerve compression injury (Best et al., 2019). In the central nervous system, neuroplastic changes that involve the spinal cord and thalamus were observed in patients or experimental models (Selvarajah et al., 2014; Marshall et al., 2017). The dorsal root ganglia (DRG) contains most of cell bodies of primary sensory neurons, which transmits sensory neural signals through the peripheral nerves to the central nervous system (Maatuf et al., 2019). Due to exposure outside the blood-brain barrier, DRG is particularly vulnerable in diabetes and could be an important trigger for neuropathic pain (Sloan et al., 2018). Overall, the diversity of mechanisms determines that PDPN is difficult to cure. Current first-line treatment for PDPN is nonspecific, and serious adverse effects limit their clinical use (Todorovic, 2015; Snyder et al., 2016). Hence, deeper knowledge in molecular and cellular mechanisms of PDPN is urgently needed to provide a basis for pharmaceutical development.

Single-cell RNA sequencing (scRNA-seq) has progressed at a rapid pace for its advantages in determining tissue heterogeneity and identifying novel cell types (Rodriguez-Meira et al., 2019). Previous transcriptomic studies characterized changes that could contribute to PDPN or DPN in DRG or sural nerves (Hur et al., 2011, 2016; Yamazaki et al., 2013; Guo et al., 2020). These sequenced RNA samples were from whole tissue that inevitably contained mixed cell types. Meanwhile, the “average” gene expression profiles could possibly lead to the confounding interpretation of the results. The difference is that scRNA-seq can sequence thousands of cells in an unbiased manner to uncover both known and novel cell types. We can simultaneously classify and analyze numerous DRG neuron types to know which one is most associated with PDPN. Furthermore, thousands of cell sequencing data would help to investigate whether and how the dynamic DRG neuron type changes in response to hyperglycemia. In the current study, we performed scRNA-seq on rat DRG from PDPN models induced by streptozotocin (STZ) injection (Morrow, 2004). Our work established an unbiased classification of rat DRG neuronal types and identified a novel neuron type MAAC. MAAC was marked by Na, K -ATPase (NKA)-related genes *Fxyd7*, and *Atp1b1*. Compared with other neurons, MAAC was characterized by highly expressing genes related to neurofilaments and cytoskeletons. We inferred that it was derived from peptidergic neurons based on correlation analysis and used pseudo-time analysis to identify the gene expression kinetics in neuron type transitions. Furthermore, we studied the transcriptional regulatory networks and communication networks of MAAC. Our results provide comprehensive landscape to uncover the alteration of genes, cell types, and intercellular communication. These results advance our understanding of neuropathic pain in diabetes and provide a basis for the development of pain therapy.

## Materials and Methods

### Rat

All animal studies were approved by the Shanghai Jiao Tong University Animal Care and Use Committee and conducted in accordance with the animal policies of the Shanghai Jiao Tong University and the guidelines established by the National Health and Family Planning Commission of China. Sprague Dawley rats were obtained from Shanghai Lab Animal Research Center. Rats were housed in specific pathogen-free conditions with free access to water and rat chow.

### Diabetes

To induce diabetes, male Sprague-Dawley rats (200–220 g) were injected intraperitoneally with 60 mg/kg STZ (Solarbio, China) dissolved in 1% citrate buffer (pH = 4.5). The control group received equal volumes of the vehicle. Three days after the injections, the glucose concentration was measured in tail vein blood samples using a blood glucose meter (Bayer HealthCare, USA). Only rats with the glucose concentration higher than 11.1 mmol/l were considered diabetic (Dominguez et al., 2015). All the animals were weekly weighed and daily observed during the study.

### Mechanical Allodynia

Mechanical nociception was weekly evaluated using the von Frey filaments (North Coast, USA). Rats were separately placed in transparent plexiglass chambers on an elevated metal grid floor with 1 cm holes and probed from below. After a 15-min adaptation period, the hind paws were stimulated with varying forces (in this case 1.4, 2, 4, 6, 8, 10, 15, and 26 grams-force). Every stimulation delivered a constant pre-determined force for 5 s, and the interval of every stimulation was 15 s. The depression in the center of the rats' paws was accurately stimulated. Sensation in this area is within the innervation range of the tibial nerve, of which most fibers originate from L5 DRG in rats (Rigaud et al., 2008; Laedermann et al., 2014). The 50% paw withdrawal threshold (PWT) was determined by the up–down method of Chaplan et al. (1994). The experiment begins by testing the response to the 6 g filament. If a positive response occurred, the next filament with a lower force was applied. Conversely, if a negative response occurred, the next filament with a higher force was applied. This continues until at least four readings are obtained after different reactions appear for the first time. If the force to be used is higher than 15 g or lower than 1.4 g, the threshold on this side is directly recorded as 15 g or 1.4 g, respectively (Deuis et al., 2017). Finally, diabetic rats were divided into groups on day 28 after STZ injection. Diabetic rats with the PWT ≤ 8 g were considered to develop MA. Rats with the PWT ≥ 15 g were selected into the group without MA, and other diabetic rats were excluded from subsequent experiments.

### Tissue Dissociation and Cell Purification

In total, 24 rats (eight in each group) were sacrificed 28 days after STZ injection. Among these animals, two control group rats, four diabetic rats with MA, and two diabetic rats without MA were used for scRNA-seq. DRGs, dissected from rats of the abovementioned groups, were washed with Hanks Balanced Salt Solution (HBSS; Sigma-Aldrich, USA) three times. Then, the tissue pieces were transferred into a 15 ml centrifuge tube and digested with 2 ml GEXSCOPE^TM^ Tissue Dissociation Solution (Singleron, China) at 37°C for 15 min under sustained agitation. After digestion, the samples were filtered using 40 μm sterile strainers and centrifuged at 1,000 rpm for 5 min. The supernatant was discarded and the sediment was resuspended in 1 ml phosphate-buffered saline (PBS; HyClone, USA). Two milliliters of GEXSCOPE^TM^ red blood cell lysis buffer (Singleron, China) was added to remove the red blood cells for 10 min at 25°C. The solution was then centrifuged at 500 × g for 5 min and suspended in PBS. The sample was stained with trypan blue (Sigma-Aldrich, USA) and microscopically evaluated.

### Library Construction and Sequencing

The scRNA-seq libraries were constructed following the protocol of GEXSCOPE^TM^ Single-Cell RNA Library Kit (Singleron, China) (Dura et al., 2019). Briefly, the single-cell suspensions were loaded onto microfluidic devices. Then, millions of beads with unique cell barcodes and unique molecular identifiers (UMIs) were loaded similar to cells. Only one bead was loaded in each microwell. Following lysis, mRNA was captured onto the beads. More than 95% of the beads were recovered, and the captured mRNA was reverse-transcribed. After cDNA amplification and enrichment, the resulting scRNA-seq libraries were sequenced on Illumina HiSeq X10 instrument with 150 bp paired end reads. Sequencing data of cell populations was conserved in the National Center for Biotechnology Information (NCBI) database with the Gene Expression Omnibus (GEO) accession number GSE176017.

### Pre-Processing of scRNA-Seq Data

The raw reads were processed to generate gene expression profiles by an internal pipeline, namely, FastQC for quality evaluation, fastp for trimming, STAR aligner (2.5.3a) for alignment, and featureCounts (1.6.2) for transcript counting (Dobin et al., 2013; Liao et al., 2014; Chen et al., 2018; Wingett and Andrews, 2018). Briefly, after filtering read 1 without polyT tails, cell-barcode and UMI were extracted. Adapters and polyA tails were trimmed before read 2 was mapped to RGSC rn6 reference genome with ensemble version 92 gene annotation (http://www.ensembl.org). Reads with the same cell barcode, UMI, and gene were grouped to calculate the number of UMIs per gene per cell. UMI count tables of each cellular barcode were used for further analysis.

### Data Integration and the Dimensionality Reduction

The data were processed by the Seurat (version 4. 0. 4) (Hao et al., 2021) in R software (version 4. 1. 1). According to quality control metrics (Ilicic et al., 2016), cells with genes >2,500 or <200 and the cells that have >10% mitochondrial genes were filtered out. A total of 6,693 cells that were obtained (2,543 in the control group, 1,192 in diabetic rats without MA, and 2,958 in diabetic rats with MA) were used for further bioinformatics analysis. Gene expression of each cell was normalized by total expression, multiplied by a scale factor of 10,000, and log-transformed (NormalizeData function). The 2,000 highly variable genes (HVGs) were selected (FindVariableGenes). Then, we integrated different samples (IntegrateData), and the technical or batch effect was eliminated by canonical correlation analysis (CCA). The expression of each gene that was scaled to shift its mean/variance across cells is 0 and 1 (ScaleData). These results were used as input for dimensionality reduction *via* principal component analysis (PCA).

### Unsupervised Clustering and Cell Type Identification

The top 30 principal components were chosen for cell clustering. The clustering analysis was performed based on FindClusters function after building the nearest neighbor graph using FindNeighbors function. The main cell clusters were identified and visualized with t-distributed stochastic neighbor embedding (t-SNE) plots or uniform manifold approximation and projection (UMAP) plots. Due to differences in algorithms, the distance between two points in the two-dimensional plane of UMAP plots can represent the difference in gene expression information between two cells of the high-dimensional space better. To annotate the cell clusters, cluster biomarkers with high discrimination abilities were identified (FindMarkers function). The cell groups were annotated based on SingleR (Aran et al., 2019) and conventional markers described in previous studies (Fallon, 1985; Arroyo et al., 1998; Le et al., 2005; Dhaka et al., 2007; Schroeter and Steiner, 2009; Usoskin et al., 2015; Li et al., 2016; Oikari et al., 2016; Urban-Ciecko and Barth, 2016; Wu et al., 2017; Donovan et al., 2018; Hockley et al., 2019; Ronning et al., 2019; Zhang et al., 2019; Avraham et al., 2020; Gerber et al., 2021). Similar procedures were applied in the subclusters identification of neurons.

### Enrichment Analysis

The gene ontology (GO) enrichment analysis was explored in Database for Annotation, Visualization, and Integrated Discovery (DAVID) (Ashburner et al., 2000).

### Pseudo-Time Trajectory Analysis

To discover the gene expression kinetics in neuron type transition in PDPN development, the pseudo-time trajectories were generated with the Monocle package (version 2.18.0) (Trapnell et al., 2014). It can arrange cells along the pseudo-time trajectory, where each cell corresponds to a distinct time point, showing their developmental trajectories such as cell differentiation and other biological processes. The gene expression matrix derived from the Seurat processed data were used as the inputs. The differentially expressed genes (DEGs) were identified (*q*-value < 0.1) to sort cells in a pseudo-time order. Dimension reduction was performed using the DDRTree method. DEGs were clustered into different categories according to the gene expression patterns along the pseudo-time (Plot_pseudotime_heatmap function). Biological processes were revealed using GO enrichment analysis in DAVID.

### Transcription Factor Inference

The analysis of the single-cell gene regulatory network was performed using the single-cell regulatory network inference and clustering (SCENIC) package (Aibar et al., 2017). Rat gene symbols were converted in the corresponding mouse homologous genes using the homologene R package (github.com/oganm/homologene; www.ncbi.nlm.nih.gov/homologene). After initializing settings, the expression matrix was loaded onto GENIE3 for building the initial co-expression gene regulatory networks (GRN). The regulon data were analyzed using the RcisTarget package and the network activity was evaluated. The transcriptional network of TF and predicted target genes were visualized by Cytoscape (Shannon et al., 2003).

### Cell Communications Analysis

Cell communications were analyzed based on CellChat (Jin et al., 2021). The ligand-receptor interaction database can be found at http://www.cellchat.org/. All the rat gene names were converted to the mouse ortholog using the homologene R package. The communication network was generated to discover the number of ligand and receptor (L-R) pairs and the signaling strength between each cell cluster. The strength was defined by the communication probability, which was calculated by a specific algorithm using the geometric mean of the normalized expression of receptors and ligands as input data. Outgoing and incoming interaction strength was calculated to compare the communication ability of each neuronal cluster as senders and receivers. The communication probabilities mediated by L-R pairs were compared and visualized in the bubble plot.

### Statistical Analysis

All statistical analyses and the graph generation were performed in R (version 4. 1. 1).

### Code Availability

The analysis codes are available on GitHub: https://github.com/SJTU-ZhouHan/SJTU-ZhouHan.

## Result

### Cellular Constitution of Rat DRG

**Figure 1:**
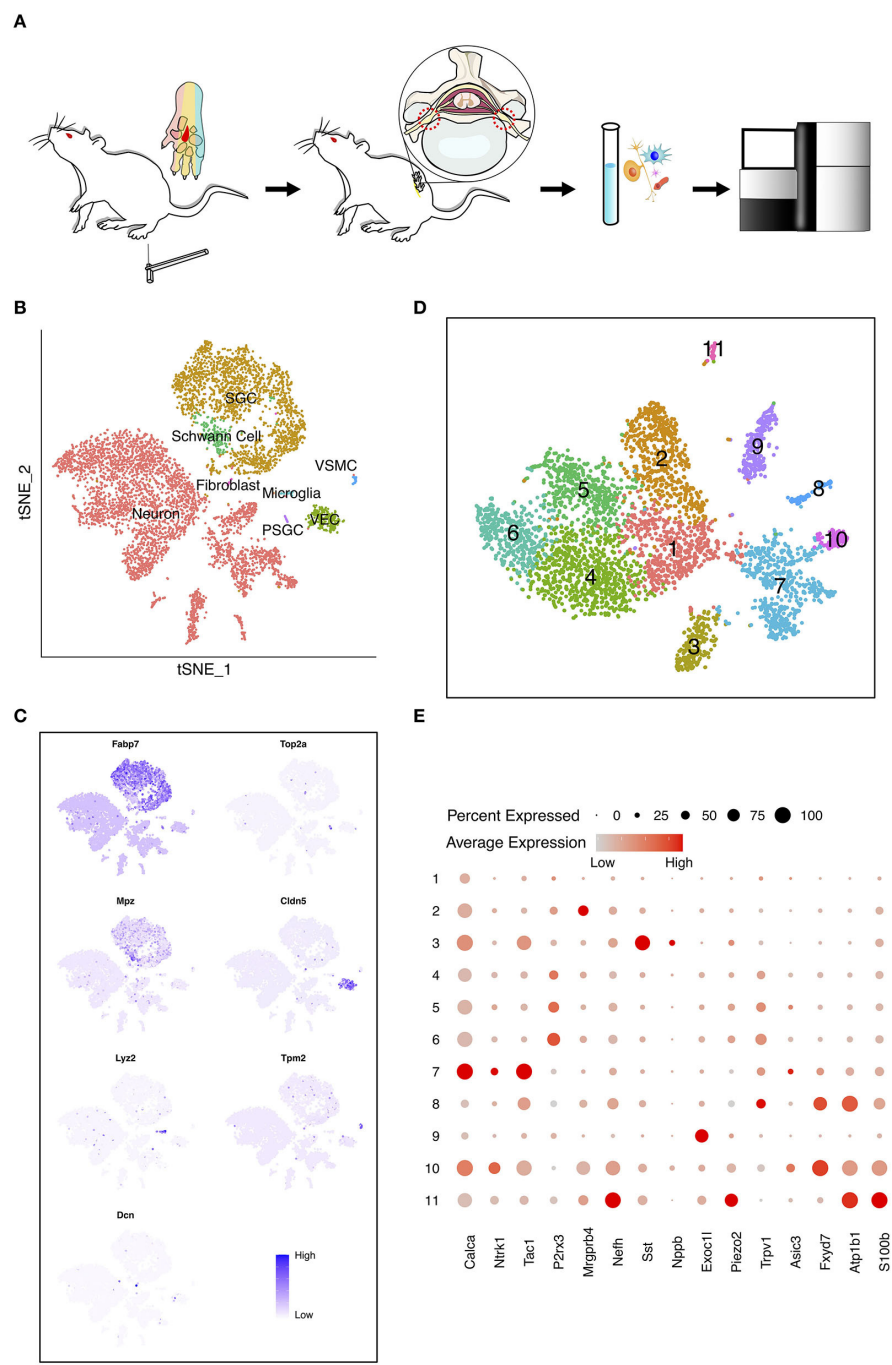
The heterogeneity of dorsal root ganglia (DRG) cells in the painful diabetic peripheral neuropathy (PDPN) model. **(A)** Workflow of the pain threshold evaluation, sample preparation, sequencing, and bioinformatic analysis. The red region in the center of the rats' paws was stimulated by von Frey filaments. Sensation in this area is within the innervation range of the tibial nerve, originating from L5 DRG in rat. **(B)** t-distributed scholastic neighbor embedding (t-SNE) plot of single cells profiled in the presenting work colored by cell types. Each colored dot represents a cell. **(C)** Feature heatmap shows the marker genes in each cell type. The color represents the gene expression level after batch effect correction and normalization. **(D)** t-SNE plot shows the unsupervised clustering DRG neurons. Dots, individual cells; colors, neuron clusters. **(E)** Dot plot shows the differentially expressed genes (DEGs) of each DRG neuron cluster. The size of the dot means the percentage of cells expressing the gene, and the color indicates the average expression level.

In the present work, we intended to investigate the cell diversity of DRG neurons in the adult rat under PDPN conditions. A conspicuous MA typically becomes steady 4 weeks after diabetes induction in rats. We performed scRNA-seq on the cells dissociated from bilateral lumbar (L) 5 DRGs of diabetic rats with and without MA and normal control groups (Figure 1A; Supplementary Figure 1). After quality control, 6,693 cells, including 3,979 neurons, were obtained. Visualization of single-cell transcriptomes in t-SNE space was able to separate cells into clusters which we mapped to eight major cell types with distinct markers (Figure 1B). These cells included neurons, satellite glial cells (SGC), proliferating SGC (PSGC), Schwann cells, fibroblasts, vascular smooth muscle cells (VSMC), vascular endothelial cells (VEC), and microglia. SGC were identified due to highly expressed *Fabp7* (Figure 1C) (Goncalves et al., 2017). PSGC specifically expressed *Fabp7* and proliferation markers *Top2a* (Figure 1C) (Gerber et al., 2021). The cell population that highly expressed *Mpz* was annotated as Schwann cells (Figure 1C) (Le et al., 2005). Microglia highly expressed *Lyz2* (Figure 1C) (Donovan et al., 2018; Ronning et al., 2019). In addition, we identified VEC marked by *Cldn5* and VSMC by *Tpm2* (Figure 1C) (Jang et al., 2011; Kalluri et al., 2019).

### Subclusters-Specific Analysis of Neurons

**Figure 2:**
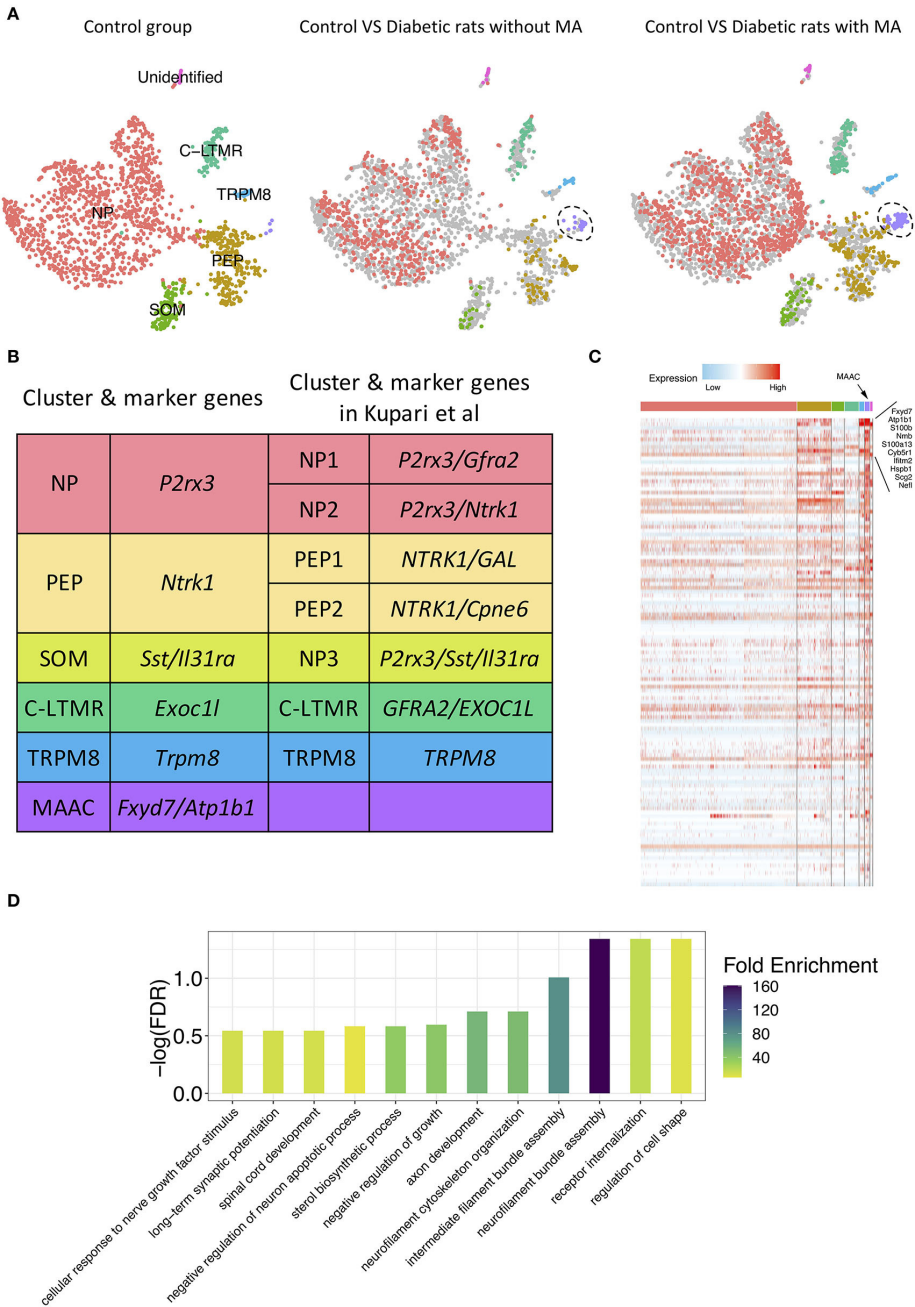
Novel neuron cluster mechanical allodynia-associated cluster (MAAC) appeared in the development of PDPN. **(A)** t-SNE plot shows somatosensory neuron clusters in control versus in diabetic rats without or with mechanical allodynia (MA). The dots in the control group are grayed out when they are compared with the dots in the other two groups. Dots, individual cells; colors, neuron clusters. **(B)** Summary of neuron type classification in this work and previous study. **(C)** Heatmap shows the DEGs of MAAC. The genes with the largest fold difference are marked. Color, expression level. **(D)** Bar plot shows gene ontology (GO) terms of biological processes [false discovery rate (FDR) <0.3] enriched for the DEGs of MAAC.

To detect discrete neuron subclusters, we reclassified neurons based on the canonical DRG neuron markers (Usoskin et al., 2015; Liguz-Lecznar et al., 2016; Kupari et al., 2021). Eleven neuron clusters and their DEGs were unbiasedly identified by Seurat program (Figures 1D,E). These clusters were annotated as non-peptidergic nociceptors (NP), peptidergic nociceptors (PEP), somatostatin-positive neurons (SOM), C-fiber low-threshold mechanoreceptors (C-LTMR), and *Trpm8*-positive neurons (TRPM8) (Figure 2A). We identified MA-associated clusters (MAAC) for the obviously increased cell number in diabetic rats with MA (Figure 2A). The specific neuron number for each cluster is given in Supplementary Table 1. Neuron clusters were matched with a previous scRNA-seq study of macaques DRG (Figure 2B) (Kupari et al., 2021). NP was named based on Purinergic receptor P2X3 (*P2rx3*), and PEP was named based on tropomyosin receptor kinase A (TRKA, *Ntrk1*). SOM was identified using somatostatin (*Sst*) and interleukin 31 receptor A (*Il31ra*). Although SOM contains markers for both peptidergic and nonpeptidergic neurons (*Tac1* and *P2rx3*), this cluster was named NP3 in previous literature (Usoskin et al., 2015). *Exoc1l, P2ry1*, and *Gfra2* were used to assign C-LTMR. *Trpm8* expression was used for naming the TRPM8 neuron cluster.

### Emerging Novel Neuron Cluster Associated With MA

The t-SNE plot was split based on different groups to present the progression of the emerging novel neuron cluster MAAC (Figure 2A). Neurons in other clusters were evenly distributed in different groups (Figure 2A). Hundreds of DEGs of MAAC were identified (log2FC > 0.25 and adj. *p*-value < 0.05) (Figure 2C; Supplementary Table 2). Among these genes, *Fxyd7* and *Atp1b1* were highly expressed in MAAC. The paralog of *Fxyd7, Fxyd1*, and the paralog of *Atp1b1* and *Atp2b4* were also included (Supplementary Table 2). We next annotated the DEGs with biological processes [false discovery rate (FDR) < 0.3] from GO databases to obtain transcriptomic characteristics of MAAC. These cell-type specific genes recapitulated neurofilament, axon, synapse, and receptor-related biological processes (Figure 2D; Supplementary Table 3). “Regulation of cell shape,” “receptor internalization,” and “neurofilament bundle assembly” were the most significant enrichment terms. “Neurofilament bundle assembly” was the most enriched biological process with the highest fold enrichment.

### The Origin and Transition of MAAC

**Figure 3:**
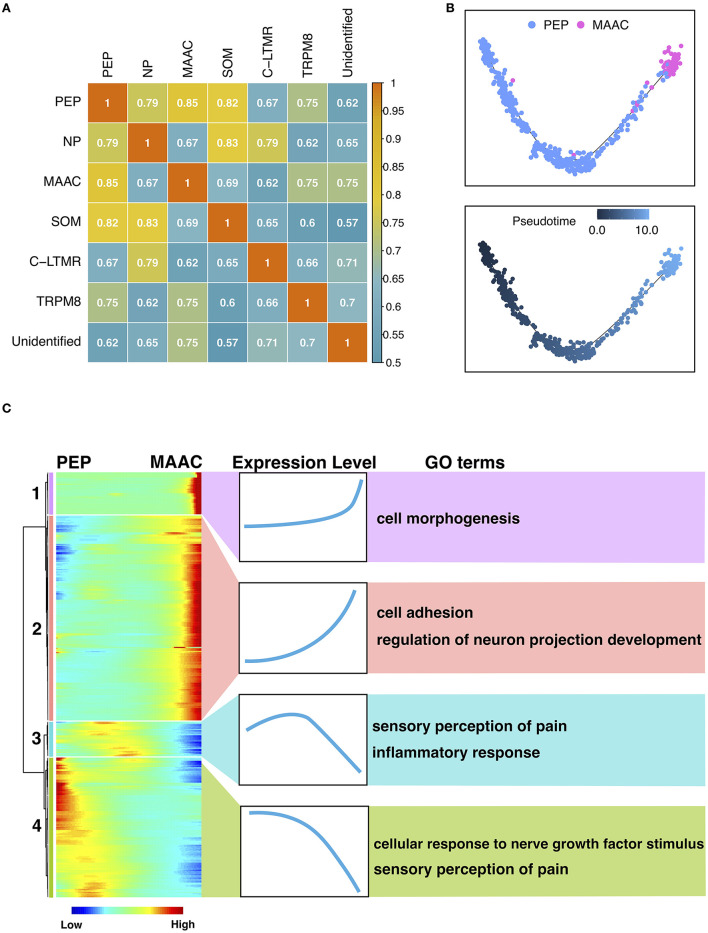
The origin and transition of MAAC. **(A)** Heatmap shows the Pearson correlation of each neuron cluster based on their genes expression files. **(B)** Pseudo-time trajectories show the fate of MAAC originated from peptidergic nociceptors (PEP). Dot, cells; colors, neuron clusters or pseudotime. **(C)** Heatmaps show the DEGs clustered based on their dynamic expression characteristic, which were shown in pseudo-time from PEP to MAAC.

To explore the origin of MAAC, we calculated the transcriptomic correlations of different neuron clusters. The heatmap showed that the gene expression of MAAC was close to PEP (Figure 3A), and the distance between these two clusters on the UMAP plot also provides corroborative evidence (Supplementary Figure 2). Then, we reconstructed the pseudo-time trajectories and identify genes whose expression changed as the neurons underwent transition (Figures 3B,C). Genes in the neuronal switch process from PEP to MAAC were classified into four modules based on their expressing patterns (Figure 3C). GO enrichment analysis were used to analyze the biological processes of each gene module. Upregulated genes in Module 1 were associated with “cell morphogenesis,” including *Tgfb2, Tenm4, Hgf* , and *Atp2b2*. Module 2 reflected the genes that were upregulated from a lower level across pseudo-time. These genes were enriched in “cell adhesion” and “regulation of neuron projection development.” Comparatively, the expression of genes related to “inflammatory response” eventually decreased. Notably, some genes involved in “sensory perception of pain” were downregulated, including *Ndn, Trpv1, Asic1, Aqp1* (Module 3), *Adcyap1, Trpa1, Npy1r, Oprk1, Tac1* (Module 4).

### Pivotal Regulons Involved in Neuron Type Transition

**Figure 4:**
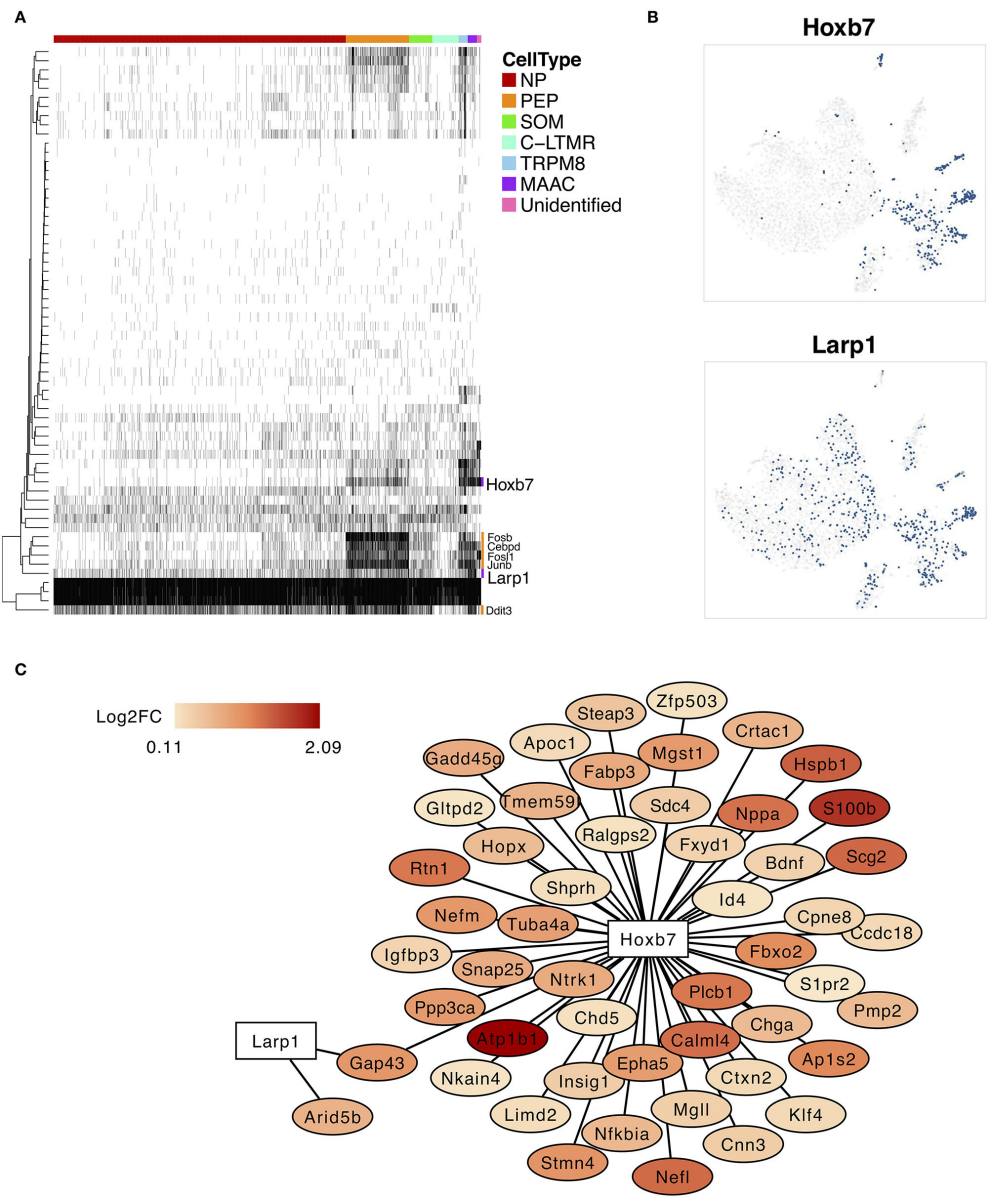
Specifically expressed transcription factors and regulatory network of MAAC. **(A)** The binary heatmap shows the activity of inferred transcription factors in different neuron clusters. **(B)** t-SNE plots show the activities cells distribution of two specific expressed regulons of MAAC. Dots, individual cells; colors, activated cells. **(C)** Gene regulatory networks of *Hobx7* and *Larp1* inferred by single-cell regulatory network inference and clustering (SCENIC). Colors indicated the log2FC of DEGs of MAAC.

The SCENIC analysis was performed to identify the critical regulators and to reconstruct gene regulatory network. The binary regulon activity matrix was generated and two MAAC-specific regulons, *Hobx7* and *Lar1p*, were identified (Figure 4A). The binary t-SNE plot showed that these TFs were expressed and activated in MAAC (Figure 4B). Other TFs like *Fosb, Cebpd, Fosl1, Junb*, and *Ddit3* had broad expression patterns. They were mainly expressed and activated in PEP and MAAC. In addition, we constructed the gene regulatory network of *Hobx7* and *Lar1p* (Figure 4C). We observed that *Hobx7* regulated the most DEGs of MAAC, including genes with high expression level, like *Atp1b1* and *S100b*. However, *Larp1* only regulated the expression of *Gap43* and *Arid5b*. Thus, *Hobx7* could serve as a critical regulator in the formation of MAAC.

### Intercellular Communication Analysis of MAAC

**Figure 5:**
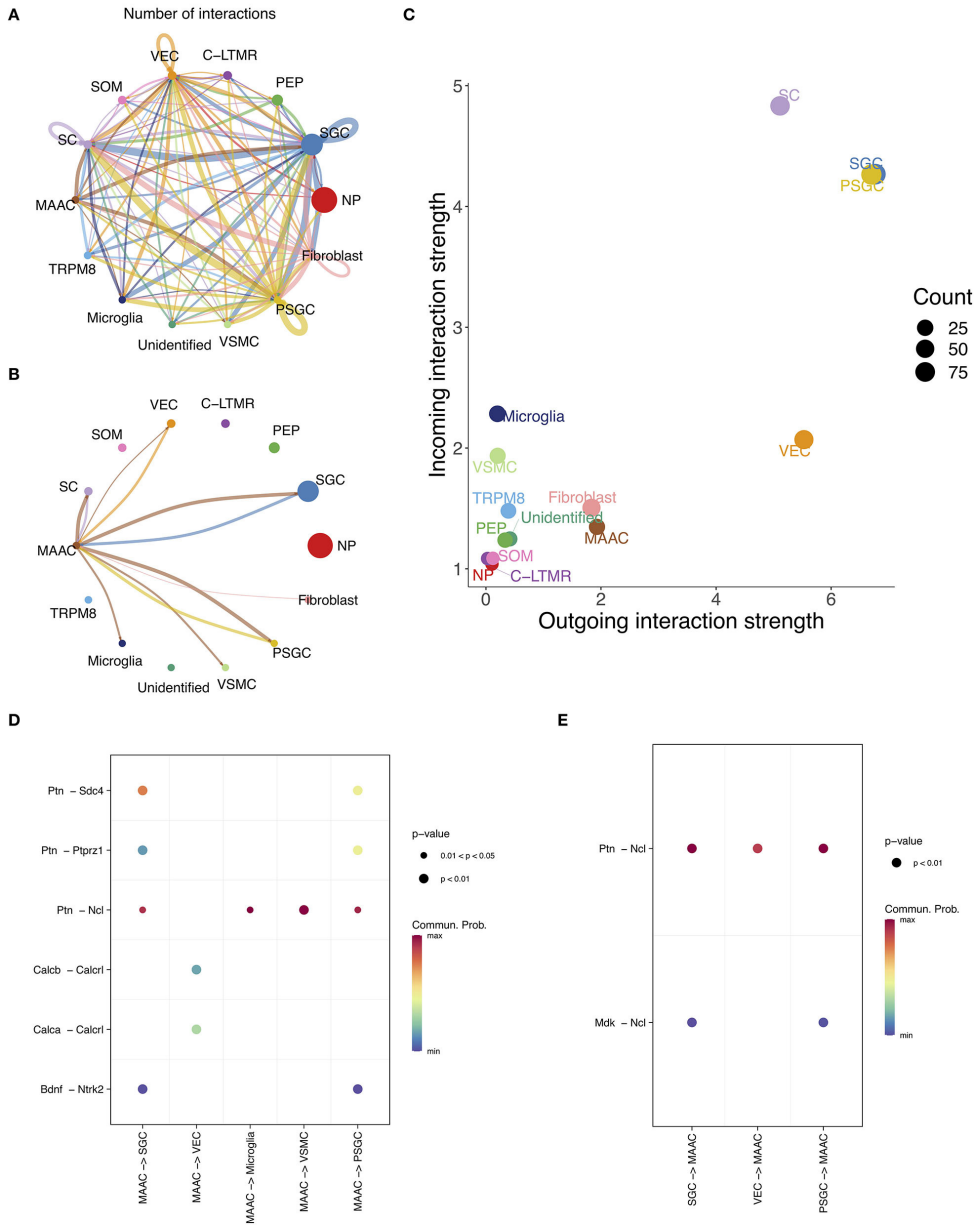
The intercellular communication of MAAC. **(A)** Intercellular communication network reflects the number of interactions between clusters. Nodes, neuronal clusters; node size, cell counts; edge width, number of interactions. **(B)** Intercellular communication network shows the number of interactions between MAAC and other neuron clusters. Nodes, neuronal clusters; node size, cell counts; edge width, number of interactions. **(C)** Dot plot shows the incoming and outgoing strength of each cell type. The outgoing /incoming interaction strength is defined by the comprehensive communication probability between the signal sending /target cells and all cell types. **(D)** Dot plot shows the communication probabilities of ligand-receptor pairs when MAAC are sender cells. Only the ligand-receptor pairs with significant changes (*p* <0.05) are shown in the picture. Commun. Prob, communication probability. The communication probability here equals to the interaction strength and is not exactly a probability. Dot color means communication probabilities and dot size represents computed p-values. **(E)** Dot plot shows the communication probabilities of ligand-receptor pairs when MAAC serves as target cells.

The intercellular communication was inferred based on the information of L-R pairs in CellChat database (Jin et al., 2021). The numbers of interactions between cell clusters were calculated (Figures 5A,B). There is a close link between MAAC and SGC, but not between other neuron clusters (Figures 5A,B). Then, we compared the outgoing and incoming interaction strength of each cell type (Figure 5C). The interaction strength of glia appeared to be greater than other cells, and the strength of MAAC was obviously greater than their PEP cluster origin. To identify the ligands or receptors involved, we respectively compared the communication probabilities mediated by L-R pairs between MAAC and other cells (Figures 5D,E). Only the L-R pairs with the most pronounced changes (*p*-value < 0.05) were displayed. When MAAC served as the signal source, communication probabilities of PTN ligand were prominent. MAAC may communicate with SGC and PSGC *via Ptn-Sdc4, Ptn-Ptprz1, Ptn-Ncl*, and *Bdnf-Ntrk2*. The communication between MAAC and VEC was mediated by *Calcb-Calcrl* and *Calca-Calcrl* in low communication probabilities. Besides, MAAC could communicate with microglia and VSMC by *Ptn-Ncl*. When MAAC served as the signal target, the *Ncl* receptor was playing an important role. The biologic behavior of MAAC may be regulated by *Ptn-Ncl* and *Mdk-Ncl*.

## Discussion

Dorsal root ganglia are vulnerable in diabetes due to lack of the protection of blood-nerve barrier. Injured somatosensory neurons could be an important trigger for pain. Here, using scRNA-seq, we generated a cell-type specific transcriptome atlas and revealed the complexity of the cellular landscape inside DRG tissues. Then, we identified a PDPN-related cluster MAAC and their origin. The transcriptomic characteristics of neuron type transition and pivotal regulons were demonstrated. Finally, we constructed the intercellular communication networks and revealed associated L-R pairs.

In the current study, we used STZ-induced diabetic rats to evoke MA. The extent of nerve damage exhibited individual differences among diabetic rodent models. In detail, in the same batch, the PWT of some rats dropped below 1.4 g, while some still stayed above 15 g on Day 28 after STZ injection. We compared L5 DRG cells from mechanically sensitive (diabetes with MA; PWT ≤ 8 g), insensitive rats (diabetes without MA; PWT ≥ 15 g), and normal rats.

Traditionally, DRG neuron classification was based on size and neuron-type genetic marker, but they were not always consistent with the functional heterogeneity of DRG neurons (Murray and Cheema, 2003). In contrast, comprehensive transcriptome analysis of scRNA-seq data can classify neurons in an unbiased manner. Usoskin et al. (2015) clustered mice L4-L6 DRG neurons into PEP, NP, and neurofilament-containing and tyrosine hydroxylase-containing clusters. Subsequently, these clusters were further classified into 11 subtypes including thermosensitive, itch sensitive, low-threshold mechanosensitive, and nociceptive neurons, etc. Compared with this study, we also identified cold thermoreceptors (TrpM8), C-LTMR, and SOM neurons which expressed the itch-related biomarker Il31ra. The SOM was named NP3 in the study of Usoskin et al. (2015). Because it expressed both peptidergic and non-peptidergic neuronal markers, and the distance in space is relatively independent in the t-SNE plot, we think it is more reasonable to label it as SOM. Kupari et al. (2021) classified macaques' primate DRG sensory neurons. The results of their neuron subtypes identification were broadly consistent Usoskin et al.'s (2015). A difference was that *Nefh*^+^ neurons were not clustered into one subcluster as NF cluster in the latter, which was closer to our classification result.

In previous single-cell or single-nucleus sequencing studies of different models of pain, injury-related subpopulations of neurons have been reported (Nguyen et al., 2019; Renthal et al., 2020; Wang et al., 2021). In the present study, we found that a new neuron type appeared in the development of PDPN, namely, MAAC (*Fxyd7*^+^*/Atp1b1*^+^), and identified the transcriptomic characteristics, origin, transition, and intercellular communication of them. Two DEGs with the highest fold change were considered as the biomarkers of MAAC. Interestingly, they are both closely associated with the NKA, and the dysfunction of NKA has been reported in patients with DPN (Vague et al., 1997; Krishnan et al., 2008). *Fxyd7* proven to be a regulator of NKA isozymes, which could significantly affect the apparent affinity for extracellular K^+^ of NKA α1–β1 complexes (Béguin et al., 2002). The paralog of *Fxyd7* and *Fxyd2* could regulate neuronal activity by modulating NKA activity, which may be a fundamental mechanism underlying the persistent hypersensitivity to pain (Wang et al., 2015). *Atp1b1* encodes the protein of NKA family. Its paralog, *Atp1b3*, has been reported to be involved in the formalin pain behavior (LaCroix-Fralish et al., 2009).

The enrichment analysis investigated multiple processes of MAAC. Some processes with high significance were involved in neurofilament and cytoskeleton, including “regulation of cell shape,” “neurofilament bundle assembly,” and “neurofilament cytoskeleton organization.” The mechanisms of how the cytoskeleton affects mechanical nociception are not fully understood, but it is known that through cytoskeleton force, it can be transmitted to cell membrane receptors, making them more responsive to pressure (Ingber, 2006). Bhattacherjee et al. (2017) reported that ganglion cytoskeletal genes were critical in determining mechanosensory properties in Rett syndrome. In addition, Dina et al. (2003) suggested that inflammatory mediator-induced MA was differentially dependent on the neuron cytoskeleton and that cytoskeletal disruptors could attenuate epinephrine-induced hyperalgesia in rat paws. Thus, activated cytoskeletal genes are another important feature of MAAC.

Our results suggested MAAC could originate from PEP by the alteration of gene expression profiles. The similar cellular transition was found in spared nerve injury (SNI) models. Wang et al. (2021) found three novel neuronal clusters induced by SNI. Most of them originate from the peptidergic neuron cluster. Then, through tracing the inferred trajectories, the dynamic characteristics were revealed. Notably, some enriched genes in sensory perception of pain were downregulated during MAAC formation. A similar phenomenon was also present after SNI injury (Wang et al., 2021). Further, based on the binary heatmap of regulon activity, we found the fate of MAAC could be determined by *Hobx7* and *Larp1*. *Hobx7* played a dominant role and was involved in the regulation of many DEGs of MAAC.

Next, the cellular communication activity of MAAC was inferred, and we found abundant communication relationships between MAAC and SGC and endothelial cells and microglia. Soma of neurons could not form synaptic contact with one another (Pannese, 1981), and each soma is tightly enwrapped by SGC. Therefore, the interactions of MAAC with other neurons were not found, and how hyperglycemia influences the signaling between SGC and neurons should be of particular concern. Both MAAC and SGC can send or receive signals to or from each other *via Ptn-Ncl*. SGC can also influence MAAC *via Mdk-Ncl*. *Ncl* encodes nucleolin, which is involved in the synthesis and maturation of ribosomes (Hirano et al., 2005). Previous studies have pointed out that nucleolin in neuronal cell bodies can restrict axon growth (Perry et al., 2016). Hence, we hypothesize that SGC may regulate abnormal axon growth through *Ptn-Ncl* and *Mdk-Ncl* in PDPN.

In summary, the present study reveals the single-cell transcriptomic alterations of somatosensory neurons in PDPN, identifies a novel neuron type MAAC, and investigates the novel neuron type's transcriptomic characteristics, origin, transition trajectory, regulons, and cellular communication. Thus, the study's findings advanced our current understanding of PDPN and neuropathic pain, which can be a resource for developing pain therapy. However, there were some limitations in the present study. For example, further basic experiments are needed to validate our results, and how the novel cluster MAAC induces pain symptom remains to be further clarified.

## Data Availability Statement

The datasets presented in this study can be found in online repositories. The names of the repository/repositories and accession number(s) can be found in the article/Supplementary Material.

## Ethics Statement

The animal study was reviewed and approved by the Ethics Committee, Ninth People Hospital Affiliated to Shanghai Jiao Tong University School of Medicine.

## Author Contributions

HZ and XY built models, collected specimens, analyzed data, and wrote the manuscript. CL and HC analyzed data. YW, BX, and FM contributed to the discussion. WZ reviewed the manuscript. All authors contributed to the article and approved the submitted version.

## Funding

This work was supported by the National Natural Science Foundation of China (Grant No. 81771320).

## Conflict of Interest

The authors declare that the research was conducted in the absence of any commercial or financial relationships that could be construed as a potential conflict of interest.

## Publisher's Note

All claims expressed in this article are solely those of the authors and do not necessarily represent those of their affiliated organizations, or those of the publisher, the editors and the reviewers. Any product that may be evaluated in this article, or claim that may be made by its manufacturer, is not guaranteed or endorsed by the publisher.
