# Supplementary material for: The Development of Mechanical Allodynia in Diabetic Rats Revealed by Single-Cell RNA-Seq
Source: Front Mol Neurosci. 2022 May 20;15:856299. doi: 10.3389/fnmol.2022.856299 (PMC9165721; doi:10.3389/fnmol.2022.856299)
Supplement: Supplementary file 1 [file Data_Sheet_1.docx]

Supplementary Material

# Supplementary Figures and Tables

## Supplementary Figure 1


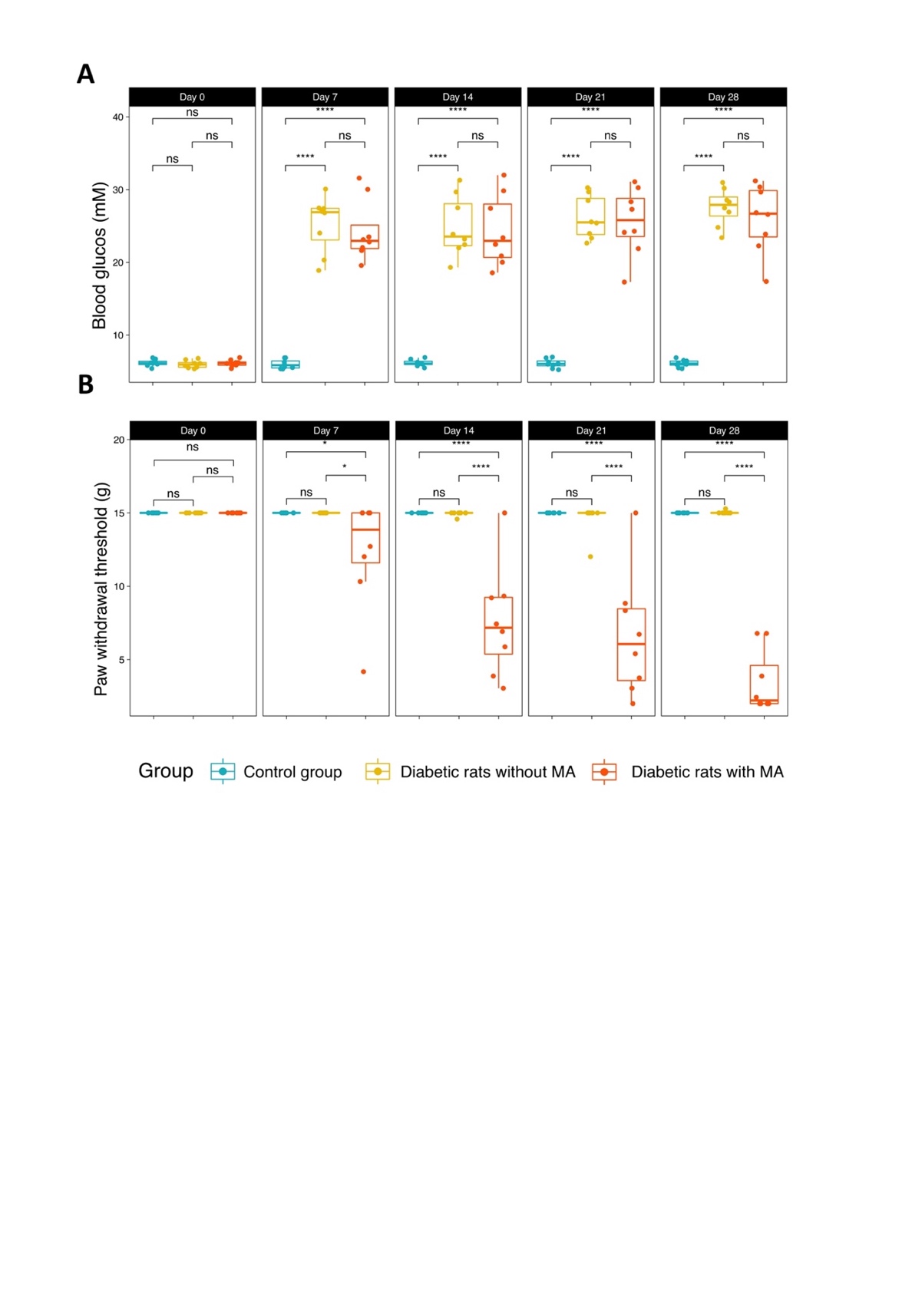


**Supplementary Figure 1.** **The development of mechanical allodynia in diabetic rats.**

The blood glucose and the 50% paw withdraw mechanical threshold were valuated every week after STZ injection.

1. Blood glucose levels of rats in different groups. (n = 8)
2. Mechanical allodynia is shown as the 50% paw withdraw mechanical threshold. (n = 8).

*,p ＜ .05; **,p ＜ .01; ***,p ＜ .001; ****,p ＜ .0001; ns, no significant.

N, normal; DM, diabetes mellitus; PDPN, painful diabetic peripheral neuropathy.

## Supplementary Figure 2

**Supplementary Figure 2.**  **UMAP plot of neuronal clusters.**

UMAP plot shows all neurons are clustered in different clusters. The Euclidean distance between MAAC and PEP is the shortest. Dots, individual cells; colors, neuron clusters.

## Supplementary Table 1

The number of neurons in different groups of each neuronal cluster. NP, non-peptidergic nociceptors; PEP, peptidergic nociceptors; SOM, somatostatin-positive neurons; C-LTMR, C-fiber low-threshold mechanoreceptors; TRPM8, Trpm8-positive neurons; MAAC, MA-associated clusters.

| Cluster | Control group | Diabetic rats without MA | Diabetic rats with MA |
| --- | --- | --- | --- |
| NP | 1213 | 525 | 980 |
| PEP | 290 | 83 | 216 |
| SOM | 128 | 23 | 65 |
| C-LTMR | 97 | 49 | 100 |
| TRPM8 | 26 | 25 | 33 |
| MAAC | 5 | 18 | 62 |
| Unidentified | 15 | 10 | 16 |
| All | 1774 | 733 | 1472 |

## Supplementary Table 2

DEGs of MAAC with log2FC > 0.25 and adj. p. value < 0.05. DEGs was calculated between MAAC and other neuronal clusters. The pct.1 means the percentage of cells in the cluster where the gene is detected. The pct.2 means the percentage of cells on average in all the other clusters where the gene is detected.

| p_val | avg_log2FC | pct.1 | pct.2 | p_val_adj | gene |
| --- | --- | --- | --- | --- | --- |
| 1.40E-62 | 2.81877666 | 1 | 0.25 | 2.79E-59 | Fxyd7 |
| 2.18E-49 | 2.09038569 | 0.965 | 0.274 | 4.35E-46 | Atp1b1 |
| 4.53E-38 | 1.74716703 | 0.965 | 0.415 | 9.06E-35 | S100b |
| 1.06E-22 | 1.64951784 | 0.812 | 0.304 | 2.12E-19 | Nmb |
| 1.50E-10 | 1.61197968 | 0.659 | 0.219 | 3.00E-07 | S100a13 |
| 1.17E-32 | 1.56601193 | 0.965 | 0.42 | 2.33E-29 | Cyb5r1 |
| 9.14E-48 | 1.56233105 | 0.918 | 0.281 | 1.83E-44 | Ifitm2 |
| 1.03E-26 | 1.41773697 | 0.965 | 0.517 | 2.07E-23 | Hspb1 |
| 7.39E-32 | 1.36039871 | 0.965 | 0.362 | 1.48E-28 | Scg2 |
| 8.17E-32 | 1.34311707 | 1 | 0.736 | 1.63E-28 | Nefl |
| 5.50E-48 | 1.31822394 | 0.871 | 0.108 | 1.10E-44 | Calml4 |
| 1.43E-32 | 1.30979241 | 0.894 | 0.281 | 2.86E-29 | Cd9 |
| 8.71E-07 | 1.282405 | 0.565 | 0.09 | 0.00174142 | Nppa |
| 8.53E-10 | 1.26911304 | 0.647 | 0.187 | 1.71E-06 | Trappc3l |
| 2.47E-26 | 1.24451361 | 0.988 | 0.75 | 4.93E-23 | Rtn1 |
| 1.59E-10 | 1.24217486 | 0.635 | 0.152 | 3.18E-07 | Plcb1 |
| 8.55E-25 | 1.12249581 | 0.929 | 0.365 | 1.71E-21 | Ap1s2 |
| 1.10E-09 | 1.07939639 | 0.671 | 0.259 | 2.21E-06 | Fbxo2 |
| 7.26E-09 | 1.06586148 | 0.6 | 0.174 | 1.45E-05 | Kcnv1 |
| 6.70E-21 | 1.0448888 | 0.976 | 0.531 | 1.34E-17 | Resp18 |
| 9.31E-42 | 1.00299139 | 0.824 | 0.188 | 1.86E-38 | Stmn4 |
| 2.01E-22 | 0.98761547 | 1 | 0.828 | 4.02E-19 | Calca |
| 3.27E-20 | 0.98601443 | 0.988 | 0.664 | 6.55E-17 | Gap43 |
| 1.47E-32 | 0.96740827 | 0.906 | 0.26 | 2.94E-29 | Snca |
| 2.37E-24 | 0.96420983 | 1 | 0.86 | 4.74E-21 | Nefm |
| 1.27E-14 | 0.96163937 | 0.647 | 0.148 | 2.55E-11 | Tmem229b |
| 8.03E-06 | 0.93951004 | 0.553 | 0.115 | 0.01606794 | Chp2 |
| 2.28E-38 | 0.93675261 | 0.871 | 0.212 | 4.56E-35 | Mgst1 |
| 2.34E-13 | 0.93617031 | 0.812 | 0.34 | 4.69E-10 | Ppp3ca |
| 4.27E-47 | 0.93214982 | 0.894 | 0.239 | 8.55E-44 | Epha5 |
| 2.35E-10 | 0.88874526 | 0.824 | 0.478 | 4.70E-07 | Ptn |
| 7.10E-17 | 0.88724885 | 0.718 | 0.201 | 1.42E-13 | Afdn |
| 4.19E-33 | 0.8858853 | 1 | 0.994 | 8.39E-30 | Calm1 |
| 4.56E-06 | 0.8799841 | 0.671 | 0.32 | 0.0091286 | Tuba4a |
| 1.17E-20 | 0.87522153 | 0.953 | 0.417 | 2.33E-17 | Tpm1 |
| 2.53E-26 | 0.86972221 | 0.871 | 0.249 | 5.05E-23 | Rbbp6 |
| 2.93E-24 | 0.82261524 | 0.682 | 0.119 | 5.87E-21 | Tdo2 |
| 3.77E-17 | 0.78364731 | 0.894 | 0.416 | 7.54E-14 | Nefh |
| 5.77E-18 | 0.77736983 | 1 | 0.827 | 1.15E-14 | Ndufa4 |
| 2.78E-12 | 0.77186178 | 0.694 | 0.259 | 5.57E-09 | Fabp3 |
| 1.53E-12 | 0.76578444 | 0.706 | 0.268 | 3.05E-09 | Ntrk1 |
| 1.87E-07 | 0.75304039 | 0.659 | 0.242 | 0.00037306 | Gadd45g |
| 1.44E-14 | 0.7469353 | 0.976 | 0.622 | 2.89E-11 | Snap25 |
| 1.32E-34 | 0.7434818 | 0.906 | 0.23 | 2.63E-31 | Prpf40b |
| 9.13E-16 | 0.73262081 | 1 | 0.817 | 1.83E-12 | Map1b |
| 1.25E-06 | 0.6936637 | 0.624 | 0.231 | 0.00250856 | Arid5b |
| 1.58E-05 | 0.68225717 | 0.6 | 0.197 | 0.03164428 | Tmem59l |
| 1.05E-10 | 0.66481302 | 0.612 | 0.176 | 2.10E-07 | Crtac1 |
| 5.23E-11 | 0.65995049 | 0.694 | 0.297 | 1.05E-07 | Tyrp1 |
| 8.75E-13 | 0.65835349 | 0.671 | 0.213 | 1.75E-09 | Palmd |
| 2.73E-06 | 0.64927732 | 0.576 | 0.19 | 0.00546106 | Snapc2 |
| 6.98E-12 | 0.62998331 | 0.906 | 0.488 | 1.40E-08 | Mdh1 |
| 3.97E-12 | 0.62311438 | 1 | 0.638 | 7.94E-09 | Kifap3 |
| 1.01E-10 | 0.61702185 | 0.671 | 0.204 | 2.03E-07 | Nfkbia |
| 3.00E-13 | 0.61602526 | 0.647 | 0.192 | 5.99E-10 | Nmt2 |
| 1.89E-11 | 0.6149938 | 0.282 | 0.055 | 3.79E-08 | S100a16 |
| 1.11E-13 | 0.6098363 | 0.259 | 0.114 | 2.22E-10 | Whrn |
| 1.19E-40 | 0.59539499 | 0.8 | 0.195 | 2.39E-37 | Pmp2 |
| 3.20E-06 | 0.59281157 | 0.624 | 0.234 | 0.00640348 | Chga |
| 1.28E-19 | 0.57683188 | 0.835 | 0.272 | 2.57E-16 | Hopx |
| 9.29E-09 | 0.55513323 | 0.259 | 0.116 | 1.86E-05 | Filip1 |
| 8.20E-27 | 0.51633288 | 0.8 | 0.22 | 1.64E-23 | Mitd1 |
| 1.62E-47 | 0.50500946 | 0.859 | 0.201 | 3.24E-44 | Zfp105 |
| 3.05E-11 | 0.5028434 | 0.706 | 0.244 | 6.11E-08 | Cavin3 |
| 1.59E-50 | 0.50215284 | 0.835 | 0.158 | 3.18E-47 | Ezr |
| 2.14E-10 | 0.49039784 | 0.659 | 0.22 | 4.28E-07 | Steap3 |
| 1.11E-18 | 0.48984552 | 0.741 | 0.261 | 2.22E-15 | LOC102556347 |
| 2.07E-06 | 0.48728342 | 0.294 | 0.139 | 0.00413876 | Csrp1 |
| 4.27E-12 | 0.48485812 | 0.847 | 0.377 | 8.54E-09 | Ncdn |
| 1.29E-100 | 0.46977647 | 0.859 | 0.088 | 2.58E-97 | Pllp |
| 4.31E-08 | 0.46751447 | 0.941 | 0.596 | 8.62E-05 | Map7d2 |
| 1.28E-09 | 0.45421751 | 0.647 | 0.23 | 2.56E-06 | Gamt |
| 8.10E-11 | 0.45327621 | 0.259 | 0.206 | 1.62E-07 | Nrp1 |
| 1.19E-09 | 0.44606432 | 0.671 | 0.23 | 2.38E-06 | Slc39a10 |
| 7.87E-07 | 0.43540518 | 0.588 | 0.203 | 0.00157366 | Zeb2 |
| 3.61E-07 | 0.42655172 | 0.812 | 0.424 | 0.00072202 | Myo5a |
| 2.54E-06 | 0.42352589 | 0.882 | 0.482 | 0.00507184 | Susd2 |
| 1.20E-16 | 0.41408465 | 0.718 | 0.218 | 2.40E-13 | Cmip |
| 3.50E-12 | 0.41407388 | 0.906 | 0.431 | 6.99E-09 | Bcat1 |
| 1.11E-17 | 0.40495543 | 0.729 | 0.221 | 2.22E-14 | Cnn3 |
| 3.78E-86 | 0.40240978 | 0.871 | 0.121 | 7.55E-83 | Arhgap15 |
| 1.76E-12 | 0.39936169 | 0.706 | 0.25 | 3.52E-09 | Insig1 |
| 1.40E-06 | 0.39716733 | 0.294 | 0.237 | 0.00279898 | Sdc4 |
| 1.02E-49 | 0.38161709 | 0.824 | 0.188 | 2.05E-46 | Vwa1 |
| 3.08E-09 | 0.37694102 | 0.282 | 0.186 | 6.17E-06 | Mgll |
| 2.23E-26 | 0.36987527 | 0.812 | 0.232 | 4.46E-23 | Sh3gl3 |
| 1.59E-35 | 0.36846114 | 0.835 | 0.207 | 3.19E-32 | Atp2b4 |
| 3.21E-36 | 0.36657826 | 0.859 | 0.231 | 6.42E-33 | Taf7 |
| 7.92E-07 | 0.36636092 | 0.647 | 0.253 | 0.00158304 | Cplx1 |
| 7.18E-06 | 0.3606596 | 0.988 | 0.724 | 0.0143556 | Klc1 |
| 3.46E-55 | 0.35215213 | 0.859 | 0.14 | 6.92E-52 | Xdh |
| 1.84E-20 | 0.34846412 | 0.882 | 0.271 | 3.67E-17 | Bdnf |
| 8.68E-07 | 0.34359455 | 0.282 | 0.19 | 0.0017369 | Mt1 |
| 6.47E-54 | 0.34271632 | 0.824 | 0.149 | 1.29E-50 | Dhh |
| 6.90E-31 | 0.34262796 | 0.8 | 0.18 | 1.38E-27 | Pip5k1b |
| 3.24E-06 | 0.33937871 | 0.341 | 0.276 | 0.0064857 | Fxyd1 |
| 4.80E-19 | 0.33288952 | 0.741 | 0.218 | 9.60E-16 | Igfbp5 |
| 2.49E-46 | 0.332038 | 0.835 | 0.219 | 4.98E-43 | Ak5 |
| 5.86E-20 | 0.32986681 | 0.741 | 0.214 | 1.17E-16 | Mt2A |
| 2.62E-28 | 0.32540905 | 0.765 | 0.202 | 5.24E-25 | Igfbp3 |
| 1.60E-32 | 0.31370634 | 0.824 | 0.178 | 3.19E-29 | Dnajb4 |
| 2.30E-32 | 0.31126372 | 0.859 | 0.203 | 4.61E-29 | LOC100909712 |
| 2.76E-17 | 0.30998358 | 0.812 | 0.271 | 5.51E-14 | Cpne8 |
| 3.81E-137 | 0.30426734 | 0.859 | 0.059 | 7.63E-134 | F12 |
| 5.21E-20 | 0.30246196 | 0.871 | 0.369 | 1.04E-16 | ENSRNOG00000034161 |
| 2.35E-10 | 0.28532421 | 0.294 | 0.091 | 4.69E-07 | Tmie |
| 3.85E-24 | 0.28326627 | 0.729 | 0.181 | 7.69E-21 | Snx18 |
| 3.14E-13 | 0.27514381 | 0.6 | 0.097 | 6.28E-10 | Ccdc18 |
| 1.74E-39 | 0.27235425 | 0.8 | 0.2 | 3.48E-36 | Gbp7 |
| 1.12E-37 | 0.27229681 | 0.8 | 0.206 | 2.25E-34 | Eef2k |
| 2.81E-09 | 0.27069663 | 0.612 | 0.202 | 5.62E-06 | Pcdh9 |
| 2.67E-15 | 0.26998369 | 0.659 | 0.172 | 5.34E-12 | Rell1 |
| 8.53E-07 | 0.26551961 | 1 | 0.992 | 0.00170688 | Stmn2 |
| 6.00E-15 | 0.26517502 | 0.812 | 0.279 | 1.20E-11 | Msmo1 |
| 2.20E-18 | 0.26508221 | 0.659 | 0.13 | 4.41E-15 | Slc8b1 |
| 5.50E-40 | 0.2646927 | 0.8 | 0.149 | 1.10E-36 | Klf4 |
| 2.38E-35 | 0.26456338 | 0.612 | 0.05 | 4.77E-32 | Cttnbp2 |
| 5.47E-07 | 0.2632152 | 0.294 | 0.195 | 0.00109441 | Gria2 |
| 1.69E-06 | 0.26219545 | 0.294 | 0.117 | 0.00337678 | Ctxn2 |
| 1.30E-25 | 0.2621408 | 0.718 | 0.144 | 2.60E-22 | AABR07009106.1 |
| 3.44E-09 | 0.25932556 | 0.282 | 0.056 | 6.87E-06 | Lsp1 |
| 3.99E-54 | 0.25918641 | 0.8 | 0.127 | 7.98E-51 | Apoc1 |
| 1.18E-07 | 0.25507582 | 0.294 | 0.249 | 0.00023682 | Gstm1 |

## Supplementary Table 3

Enrichment results of Gene Ontology (GO) biological processes using DAVID. The cutoff is FDR < 0.3.

| GO | Term | Count | Genes | Fold_Enrichment | FDR |
| --- | --- | --- | --- | --- | --- |
| GO:0033693 | neurofilament bundle assembly | 3 | Nefl, Nefm, Nefh | 160.8715596 | 0.04557122 |
| GO:0031623 | receptor internalization | 5 | Gria2, Calca, Cd9, Ezr, Snca | 19.15137615 | 0.04557122 |
| GO:0008360 | regulation of cell shape | 7 | Palmd, Tpm1, S100a13, Ptn, Arhgap15, S100b, Ezr | 8.937308869 | 0.04557122 |
| GO:0045110 | intermediate filament bundle assembly | 3 | Nefl, Nefm, Nefh | 80.43577982 | 0.09837652 |
| GO:0061564 | axon development | 3 | Gap43, Nefm, Nefh | 53.62385321 | 0.19460891 |
| GO:0060052 | neurofilament cytoskeleton organization | 3 | Nefl, Nefm, Nefh | 48.26146789 | 0.19460891 |
| GO:0045926 | negative regulation of growth | 3 | Mt2a, Igfbp5, Mt1 | 40.21788991 | 0.25351489 |
| GO:0016126 | sterol biosynthetic process | 3 | Cyb5r1, Insig1, Msmo1 | 37.12420607 | 0.26152876 |
| GO:0043524 | negative regulation of neuron apoptotic process | 6 | Ntrk1, Nrp1, Bdnf, Nefl, Mt1, Snca | 6.109046568 | 0.26152876 |
| GO:1990090 | cellular response to nerve growth factor stimulus | 4 | Ntrk1, Calca, Bdnf, Stmn2 | 12.86972477 | 0.28609613 |
| GO:0060291 | long-term synaptic potentiation | 4 | Snap25, Ptn, S100b, Snca | 12.61737723 | 0.28609613 |
| GO:0021510 | spinal cord development | 4 | Nefl, Nefm, Ptn, Nefh | 12.37473536 | 0.28609613 |
